# Supplementary material for: Inactivity and Ca2+ signaling regulate synaptic compensation in motoneurons following hibernation in American bullfrogs
Source: Sci Rep. 2022 Jul 8;12:11610. doi: 10.1038/s41598-022-15525-8 (PMC9270477; doi:10.1038/s41598-022-15525-8)
Supplement: Supplementary file 1 — Supplementary Figures. [file 41598_2022_15525_MOESM1_ESM.docx]

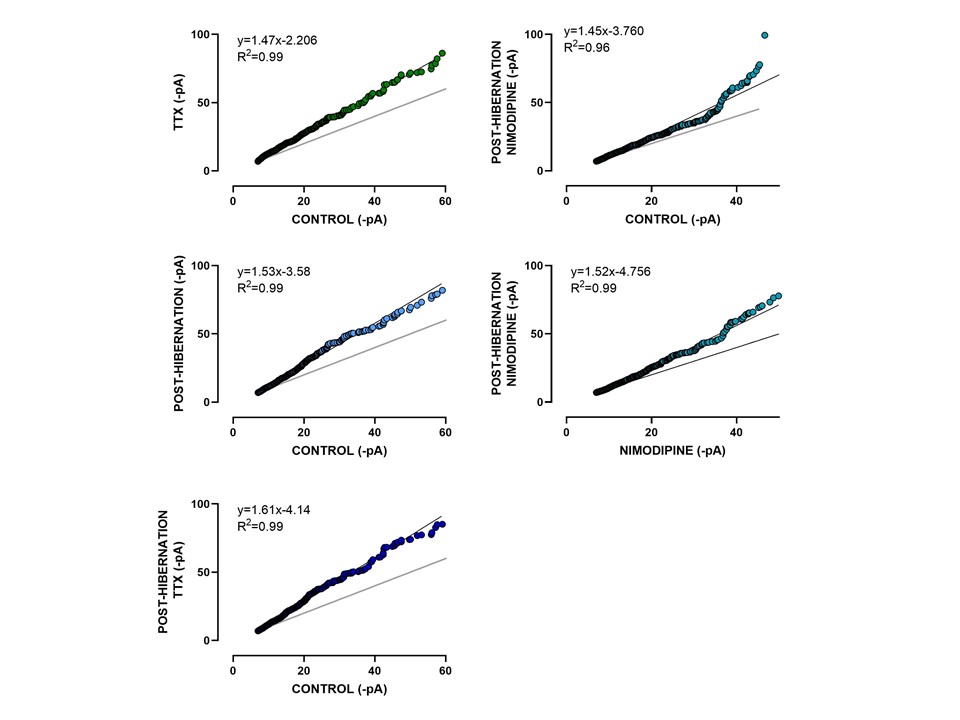


**Supplementary Figure 1 Linear fits of ranked mEPSC data for each treatment group.** mEPSCs were ranked for control (x-axis) and all treatment distributions (y-axis), plotted against each other, and fit with a linear regression. The equation was used to mathematically downscale treatment distributions to test for multiplicative scaling. Grey line in each plot shows the unity line.


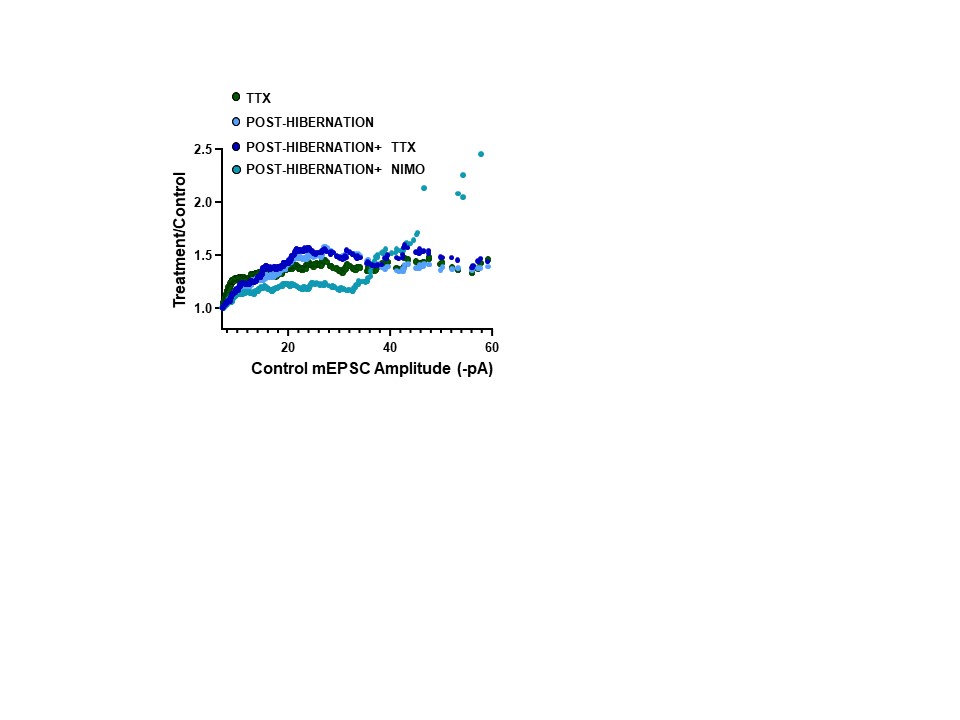


**Supplementary Figure 2. Control and treatment mEPSCs were ranked, and the ratio of “treatment/control” is plotted as a function of the control mEPSC amplitude.**
